# Effect of Sperm DNA Fragmentation and Chromatin Decondensation on PLCζ Efficacy in Infertile Patients

**DOI:** 10.3390/cimb47090707

**Published:** 2025-09-01

**Authors:** Soukaina Azil, Ismail Kaarouch, Debbie Montjean, Marie-Hélène Godin Pagé, Rosalie Cabry, Noureddine Louanjli, Bouchra Ghazi, Moncef Benkhalifa

**Affiliations:** 1 Immunopathology-Immunotherapy-Immunomonitoring Laboratory, Faculty of Medicine, Mohammed VI University of Sciences and Health, Casablanca 82403, Morocco; bghazi@um6ss.ma; 2 In Vitro Fertilization Center IRIFIV, Iris Clinic, Laboratory of Medical Analysis and Reproductive Biology, Labomac, Casablanca 20100, Morocco; nlouanjli@gmail.com; 3 African Fertility Clinic, Private Clinic of Human Reproduction and Endoscopic Surgery, Casablanca 20000, Morocco; ismail.fsr@gmail.com; 4 Fertilys Fertility Centers Laval and Brossard, 1950 Maurice-Gauvin Street, Laval, QC H7S 1Z5, Canada; debbie.montjean@fertilys.org (D.M.); marie.heleine@fertilys.org (M.-H.G.P.); 5 Reproductive Medicine, Reproductive Biology and Genetics, Peritox Laboratory, University Hospital and School of Medicine, Picardie University Jules Verne, 80054 Amiens, France; cabry.rosalie@chu-amiens.fr (R.C.); benkhalifa.moncef@chu-amiens.fr (M.B.); 6 Mohammed VI International University Hospital, Bouskoura 27182, Morocco; 7 Mohammed VI Center for Research and Innovation (CM6RI), Rabat 10112, Morocco

**Keywords:** PLCζ, DNA fragmentation, chromatin decondensation, fertilization failure, semen parameters

## Abstract

This study aimed to describe phospholipase C zeta (PLCζ) deficiency from patients who experienced oocyte fertilization failure following intracytoplasmic sperm injection (ICSI) and to investigate the relationship between sperm DNA fragmentation, chromatin decondensation, and PLCζ. A total of 135 patients participated in this study—65 fertile men and 70 infertile patients— and semen samples were obtained to analyze concentration, motility, and morphology. PLCζ protein levels were assessed by immunofluorescence and quantitative techniques, DNA fragmentation by TUNEL essay, and chromatin decondensation by aniline blue staining. The proportion of spermatozoa presenting PLCζ was significantly lower in infertile patients (18.41 ± 18.84%) compared to fertile controls (67.31 ± 13.79%) (*p* < 0.001). A significant decrease in PLCζ protein levels was observed in infertile patients compared to fertile controls, which was the same for localization patterns for each region (acrosomal, equatorial, and combination of these regions). Significant correlations were also observed between sperm parameters and PLCζ levels, DNA fragmentation, and chromatin decondensation. Furthermore, a statistically significant correlation was detected between the percentage of spermatozoa presenting PLCζ and DNA integrity (*p* < 0.001). In summary, DNA fragmentation and chromatin decondensation are associated with alterations in the localization patterns and reduced protein levels of PLCζ, which may contribute to total fertilization failure.

## 1. Introduction

During mammalian fertilization, the spermatozoon penetrates the zona pellucida of a mature metaphase II oocyte and initiates a cascade of molecular events leading to oocyte activation. This crucial step is triggered by a sperm-specific factor, phospholipase C zeta (PLCζ), which is introduced through the oocyte cytoplasm upon gamete fusion. PLCζ is considered the primary agent responsible for inducing intracellular calcium (Ca^2+^) oscillations essential for oocyte activation, resumption of meiosis, and early embryonic development [1].

**Figure 1:**
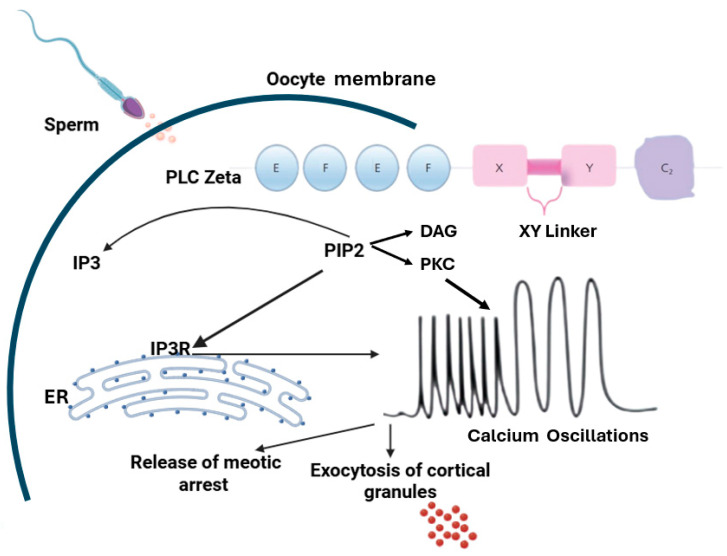
Schematic representation of the mechanisms of action of phospholipase Cζ: The PLCζ domain structure consists of four tandem EF hand domains at the N-terminus that regulate the sensitivity of the protein to calcium level. The X and Y catalytic domains at the center of the molecule cause changes in the functional ability of PLCζ to release Ca^2+^, and are separated by a short segment, the XY linker, and the C_2_ domain at the C-terminus that controls PLCζ function, and its degree of sensitivity. Oocyte activation is a consequence of several mechanisms. First, PLC zeta induces the generation of IP_3_ through the hydrolysis of PIP_2_. The generated IP_3_ binds to IP_3_R on the ER and increases the levels of Ca^2+^, resulting in the exocytosis of cortical granules and release of meiotic arrest. IP_3_, inositol 1,4,5-triphosphate; PIP_2_, phosphatidylinositol 4,5-bisphosphate; IP_3_R, inositol 1,4,5-trisphosphate receptor; ER, endoplasmic reticulum [4].

The stimulation of the phosphoinositide signaling pathway is a crucial component of the Ca^2+^ oscillations observed during mammalian fertilization, where intracellular IP_3_ and DAG are generated by the hydrolysis of PIP_2_ [2]. The generated IP_3_ then binds to its receptor in the endoplasmic reticulum, resulting in the release of Ca^2+^. The DAG produced activates the protein kinase C pathway to translate Ca^2+^ signals into cellular responses. These oscillations within the oocyte are associated with multiple processes essential for its activation, including cortical granule exocytosis, release from meiotic arrest, regulation of gene expression, recruitment of maternal mRNA, pronuclear formation, and the initiation of embryogenesis [3,4] (Figure 1).

Impairment in this signaling pathway, particularly due to deficiencies in PLCζ, is increasingly recognized as a leading cause of oocyte activation deficiency (OAD), especially in cases of fertilization failure following intracytoplasmic sperm injection (ICSI). Human spermatozoa from infertile patients may exhibit reduced or aberrant expression of PLCζ, leading to the absence or attenuation of Ca^2+^ oscillations and subsequent fertilization failure [5].

Several factors may contribute to altered PLCζ expression in spermatozoa. Notably, advanced paternal age, increased levels of sperm DNA fragmentation, and chromatin condensation defects have all been implicated in poor sperm quality and dysfunctional fertilization [6,7,8]. Recent studies have shown that DNA integrity is closely linked to sperm functionality, and high DNA fragmentation index (DFI) or sperm decondensation index (SDI) levels may negatively affect PLCζ expression, localization, and activity [9,10].

Considering these observations, the present study aimed to investigate whether abnormalities in sperm DNA integrity, specifically DNA fragmentation and chromatin decondensation, are associated with altered expression and localization of PLCζ in men experiencing fertilization failure after ICSI. Understanding this relationship could provide important insights into the molecular mechanisms underlying ICSI failure and help identify reliable biomarkers to guide clinical decision-making in assisted reproduction.

## 2. Materials and Methods

### 2.1. Study Design

This was a retrospective, case–control study conducted from January to November 2023 on 135 patients who consulted at the medical analysis laboratory and reproductive biology “LABOMAC” and the In Vitro Fertilization Center (IRIFIV) of the IRIS Clinic, Casablanca, Morocco. The study included 65 fertile controls and 70 infertile patients. For each participant, only one semen sample was collected and analyzed.

There were some inherent limitations due to its retrospective design, including potential selection bias and limited control over data collection. To minimize these biases, strict inclusion and exclusion criteria were applied, and all clinical and laboratory evaluations were standardized according to WHO 2021 guidelines [11]. Additionally, data analysis included adjustment for confounding variables such as age. Despite these efforts, prospective studies with larger samples are warranted to confirm and extend these findings.

Inclusion criteria for the infertile group were men who experienced repeated failure of oocyte activation with total fertilization failure (TFF) following at least two ICSI cycles, defined as 0% of oocytes fertilized with no 2PN observed in each cycle, while the control group consisted of fertile men with proven paternity within the past two years. To ensure that total fertilization failure (TFF) cases were primarily related to male factors, all female partners of the couples included in this study underwent thorough gynecological assessment. Only couples in which the female partner had a normal reproductive profile were included. Criteria included age below 35 years, normal AMH levels and antral follicle count, regular menstrual cycles, and no history of gynecological pathology.

All participants in this study had normal-appearing sperm parameters according to the World Health Organization (WHO) 2021 criteria guidelines, considered a criterion of selection of patients for this study [11].

Regarding clinical and demographic characteristics such as diabetes, obesity, smoking, and alcohol, these parameters were part of our exclusion criteria to minimize confounding factors that could affect sperm DNA integrity and PLCζ expression, such as a history of testicular trauma, surgery, testicular inflammation, cryptorchidism, and globozoospermia. These individuals were excluded from the study. All exclusion criteria were assessed through a standardized medical questionnaire administered at the time of recruitment, followed by a clinical andrological examination performed by a specialist. When necessary, medical records were reviewed and complementary tests were used to confirm the absence of underlying reproductive or systemic disorders.

Semen samples were collected in sterile containers by masturbating after 2 to 5 days of sexual abstinence. After the liquefaction of the sperm, we analyzed different status based on analysis of sperm quality, including sperm count ≥16 × 10^6^/mL, progressive motility ≥30%, viability ≥54%, and typical morphology ≥4% following WHO 2021 criteria [11]. For molecular assessments, PLCζ expression was evaluated by immunofluorescence staining with a specific antibody targeting PLCζ, enabling quantification of the proportion of spermatozoa expressing this key oocyte activation factor. DNA fragmentation was assessed using the TUNEL assay, while chromatin condensation status was evaluated through aniline blue staining.

### 2.2. Evaluation of Sperm Quality

Sperm samples were obtained after a period of abstinence of 2 to 5 days and left for 30 min at 35 ± 2 °C for liquefaction. Following liquefaction, all semen analyses were performed within 60 min. During this period, the samples were maintained at 37 °C on a heating stage to preserve physiological conditions. For routine analysis, ejaculate volume, pH, total sperm count in ejaculate, sperm concentration, motility, vitality, and typical morphology assessed in accordance with the World Health Organization (WHO) 2021. Sperm concentration (10^6^/mL) and progressive motility (%) were manually evaluated using a Makler counting chamber with an inverted microscope (×40) (Nikon Corporation, Tokyo, Japan). Vitality (%) assessment was performed with 2% eosin staining on a glass slide for examination under an optical microscope (×40) (Nikon Corporation, Tokyo, Japan). After centrifugation of 1 mL of the semen (800× *g* for 5 min), 20 μL of the pellet was deposited on a slide and spread-out staining with hematoxylin and Shorr solution to visualize the morphology of the spermatozoa and analyzed under an optical microscope at ×100 magnification to identify typical morphological forms following the strict criteria defined in the WHO 2021 manual [11].

### 2.3. DNA Fragmentation by TUNEL Assay

**Figure 2:**
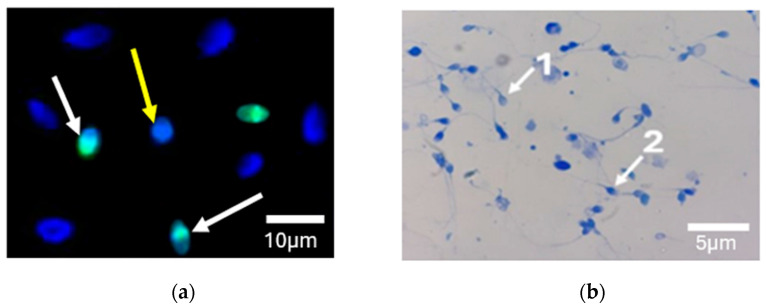
DNA fragmentation and chromatin decondensation in human sperm analyzed by TUNEL assay and aniline blue staining. (**a**) Spermatozoa with DNA fragmentation showing intense green fluorescence in the nuclear region, counterstained with DAPI to visualize nuclear morphology (white arrows), and TUNEL-negative spermatozoa stained only with DAPI, showing intact nuclear morphology (yellow arrow), observed under a fluorescence microscope (40× objective). (**b**) Chromatin decondensation assessment using aniline blue staining observed under a light microscope (100× objective). 1: spermatozoa with mature chromatin (light blue), and 2: spermatozoa with immature chromatin (dark blue). Scale bars: **a** = 10 µm; **b** = 5 µm.

The TdT-mediated dUTP nick-end labeling (TUNEL) assay was performed using an In Situ Cell Death Detection Kit (Roche Diagnostics, Mannheim, Germany) according to the manufacturer’s instructions, as previously described [12]. A volume of 1 mL of sperm was centrifuged at 800× *g* for 10 min to recover the pellet. For each patient’s spermatozoa, fractions were fixed with 4% paraformaldehyde (Merck, Darmstadt, Germany) in phosphate-buffered saline (PBS; Sigma-Aldrich, St. Louis, MO, USA) for 30 min at room temperature. Slides were then washed in PBS and permeabilized with 0.1% Triton-X 100 (Sigma-Aldrich, Merck, St. Louis, MO, USA) in 0.1% sodium citrate (Sigma-Aldrich, Merck, St. Louis, MO, USA) for 5 min at room temperature. After washing twice with PBS, slides were incubated with 5 µL of solution containing the enzyme solution and the label solution (1/10) for 45 min at 37 °C in a dark moist chamber. After two washes, slides were counterstained with Vectashield antifade medium containing 4′,6-diamino-2-phenylindole (DAPI) (Vector Laboratories, Burlingame, CA, USA). At least 100 normal spermatozoa per slide were evaluated by fluorescence microscopy (Eclipse E400, Nikon Corporation, Tokyo, Japan) ×40. Negative controls without TdT enzyme were run in each replicate. The number of spermatozoa detected with green fluorescence with a cutoff >30% is considered TUNEL-positive according to the World Health Organization guidelines [11] (Figure 2a).

### 2.4. Chromatin Condensation by Aniline Blue Staining

To perform this staining, a fresh sperm smear of each case was air-dried, fixed in 4% paraformaldehyde (Merck, Darmstadt, Germany) in phosphate-buffered saline (PBS), and then permeabilized with 0.1% Triton X-100 (Sigma-Aldrich, Merck) in 0.1% sodium citrate (Merck). Each smear was treated with aniline blue for 15 min. At least 100 spermatozoa were counted in each slide by light microscopy (Nikon Corporation, Tokyo, Japan) ×100. Unstained or light, blue-stained spermatozoa and dark-blue spermatozoa were considered normal and abnormal, respectively. A proportion of spermatozoa with dark blue-coloration greater than 30% was considered abnormal [13] (Figure 2b).

### 2.5. PLCζ Immunofluorescence Staining

Sperm samples were immunostained for PLCζ using the protocol described by Grasa et al. with some modifications [14]. At least 5 × 10^6^ spermatozoa were washed and fixed in 4% formaldehyde solution in PBS. Fixed spermatozoa were permeabilized with 0.5% Triton X-100 in PBS for 30 min. Slides were subsequently rinsed twice with PBS and incubated with PBS/5% bovine serum albumin (BSA, Sigma-Aldrich, Merck, St. Louis, MO, USA) at room temperature for one hour to block non-specific binding sites. Slides were then incubated overnight at 4 °C with 25 μg/mL of anti-human PLCζ antibody (pab0367-P, Covalab, France) and diluted in PBS/0.05% BSA. Samples were subsequently washed three times and incubated with 2 μg/mL of secondary antibody (Alexa Fluor 488 F [Ab’]2 fragment goat anti-rabbit IgG; Invitrogen, Paisley, UK) for one hour. Finally, samples were washed three times in PBS and stained with 4′-6′-diamidini-2-phenylindole (DAPI) (Vector Laboratories, Burlingame, CA, USA) on the top and covered with 20 mm × 20 mm glass coverslips for 15 min at 37 °C in a dark moist chamber. As negative controls, spermatozoa were immunostained using the same protocol, but without the primary antibody, and in a separate assay stained with only the secondary antibody to rule out any non-specific binding or background fluorescence.

**Figure 3:**
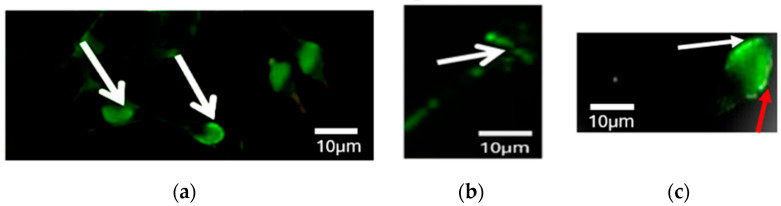
PLCζ immunostaining: presentation of different localization patterns of PLCζ in the sperm head. The sperm cells were stained with anti-PLCζ (green, **a**–**c**); (**a**) acrosomal localization (A); (**b**) equatorial localization (Eq); and (**c**): localization patterns observed included equatorial (red arrow) and acrosomal localization (white arrow) (A + Eq). Samples were observed under a fluorescence microscope (40× objective). Scale bar = 10 µm.

Samples were observed under a fluorescence microscope (Eclipse E400, Nikon) at ×40 magnification. Sperm analysis was performed with ImageJ software (version 1.52v, U.S. National Institutes of Health U.S.) using the region of interest (ROI) tool. The mean fluorescence intensity within this ROI was measured. To correct for background fluorescence, the intensity of a nearby cell-free area was measured and subtracted from the ROI intensity. For each sample, fluorescence intensity values were obtained from 200 spermatozoa and averaged to provide a representative measure of PLCζ expression. All measurements were performed under standardized conditions to ensure consistency. Only spermatozoa with the head intact and attached to the tail were selected for analysis. Spermatozoa exhibiting green fluorescence covering more than 50% of the head area were considered positive. Each analyzed spermatozoon was classified into four categories according to PLCζ localization: acrosomal (A); equatorial (Eq); acrosomal and equatorial (A + Eq); or “none,” indicating a total absence of PLCζ [15] (Figure 3).

### 2.6. Statistical Analysis

Statistical analysis was performed using the software IBM SPSS Statistics version (27.0.1.0). The Shapiro–Wilk test was used to assess the normality of data distribution prior to applying parametric tests. Student’s *t*-test was used to study the correlations between various characteristics, with two populations studied—fertile controls and infertile patients—in whom correlations between, sperm parameters, and mean levels of total PLCζ fluorescence were assessed. In order to control for the effect of age on PLCζ expression, an analysis of covariance (ANCOVA) was performed, with PLCζ expression as the dependent variable. Pearson’s correlation test was used to analyze correlation of the rate of spermatozoa expressing PLCζ between two groups. Two-way analysis of variance (ANOVA) was used to investigate the correlations between PLCζ, DNA fragmentation index (DFI), and sperm chromatin decondensation (SCD), as well as for semen parameters. All these results are expressed as means ± standard deviation (SD). Categorical data regarding the presence or absence of PLCζ localization (A, Eq and A + Eq) in fertile controls and infertile patients were analyzed using the chi-squared (χ^2^) test with contingency tables. This test was employed to compare proportions between the two groups. Statistical significance was defined as a *p*-value < 0.05.

## 3. Results

### 3.1. Comparison of Conventional Semen Parameters

The mean ages of fertile controls and infertile patients were 33.45 ± 6.43 years and 42.58 ± 6.95 years, respectively, a significant difference (*p* < 0.001). After adjustment using ANCOVA, the difference in PLCζ expression between the two groups remained statistically significant (*p* = 0.02), indicating that infertility is associated with altered PLCζ levels independently of age.

Analysis of the average values of the sperm parameters showed a significant difference between sperm count and normal sperm morphology (*p* = 0.015; *p*= 0.001, respectively), contrary to sperm motility and viability (Table 1).

### 3.2. Differences in Total Number of Sperm Exhibiting PLCζ for Fertile Controls and Infertile Patients

**Figure 4:**
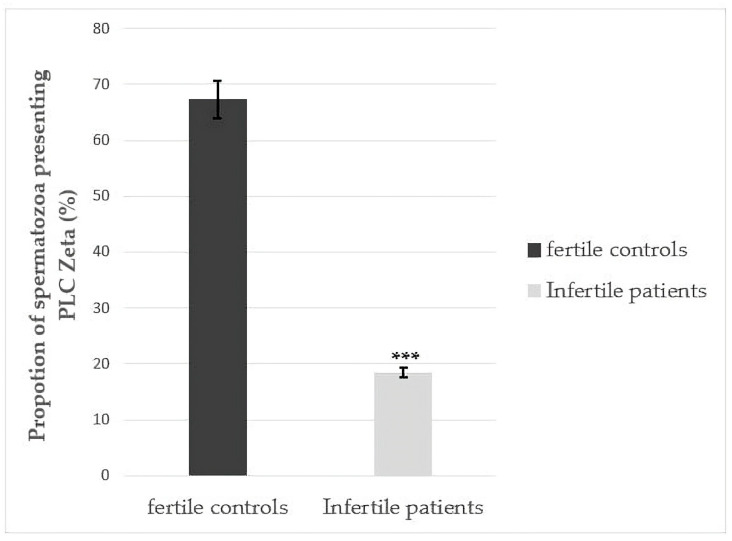
The proportion (%) of sperm exhibiting PLC zeta (means ± SD) in fertile controls and infertile patients. Statistical differences between groups were assessed using Student’s *t*-test, following confirmation of normality of data distribution. *** Significant difference (*p* < 0.001) between patients and controls.

Figure 4 shows the rate of sperm exhibiting PLCζ in fertile controls and infertile patients. A statistical significance was observed between fertile controls (n = 65) and infertile patients (n = 70) (67.31 ± 13.79% and 18.41 ± 18.84%, respectively) (*p* < 0.001).

### 3.3. Quantitative Fluorescence Analysis of PLCζ in Fertile Controls and Infertile Patients

**Figure 5:**
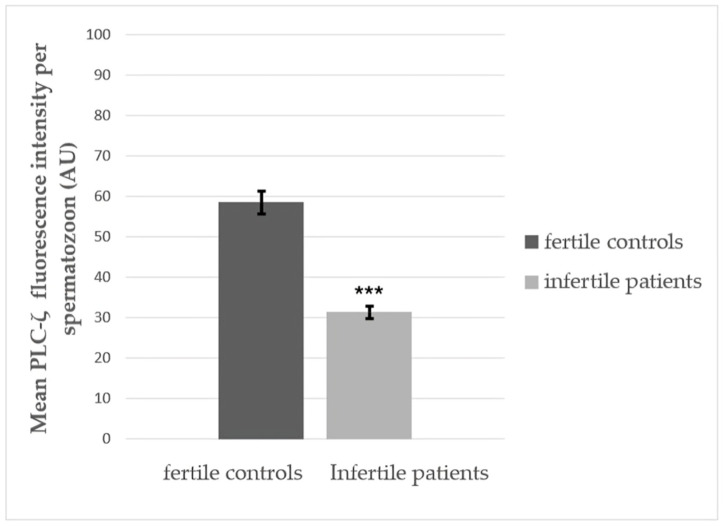
Mean relative total phospholipase C zeta (PLC-ζ) fluorescence levels exhibited by sperm from fertile controls and infertile patients. Fluorescence intensity was quantified in arbitrary units using ImageJ software. *** Highly significant difference (*p* < 0.001). Data are shown as means ± SD.

Quantitative fluorescence analysis using ImageJ software (version 1.52v, U.S. National Institutes of Health U.S.) revealed that the average PLCζ fluorescence intensity normalized to DAPI staining showed the average fluorescence intensity of PLCζ in spermatozoa from fertile controls was significantly higher (58.45 ± 24.74 AU) than recorded in infertile patients (31.32 ± 25.22 AU), presenting a significance difference (*p* < 0.001) (Figure 5).

### 3.4. Differences in PLCζ Localization Patterns in Sperm from Fertile Controls and Infertile Patients

The table below compares the presence of PLCζ protein localization patterns between fertile controls (n = 65) and infertile patients (n = 70). For acrosomal (A) localization, 50 out of 65 fertile men and 4 out of 70 infertile patients showed positive staining (χ^2^ = 70.011, df = 1, *p* < 0.001). For equatorial (Eq) localization, 16 out of 65 fertile controls and 9 out of 70 infertile patients were positive (χ^2^ = 1.951, df = 1, *p* = 0.162). For combined localization (A + Eq), 27 out of 65 fertile controls and 8 out of 70 infertile patients showed positivity (χ^2^ = 13.691, df = 1, *p* < 0.001). Statistical significance was determined using Pearson’s chi-squared test. Values with *p* < 0.05 were considered significant (Table 2).

### 3.5. Correlations Between PLCζ and Sperm Function

**Figure 6:**
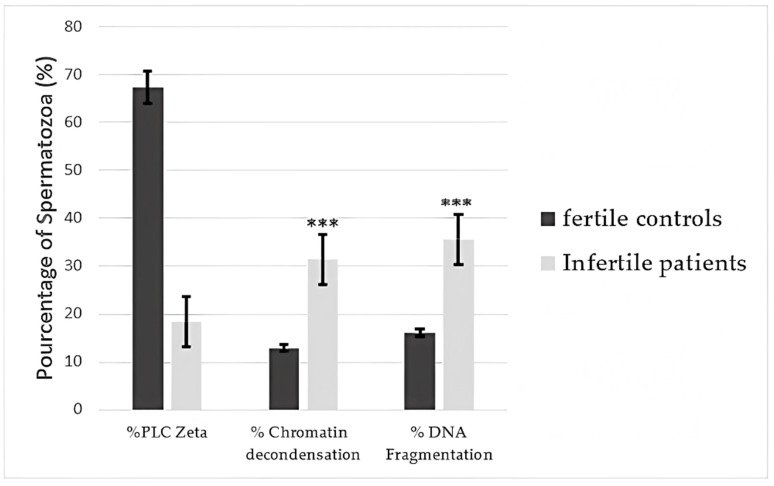
Comparison of mean percentage of spermatozoa presenting PLC zeta, DNA fragmentation, and chromatin decondensation between fertile controls and infertile patients. *** Highly significant difference (*p* < 0.001).

The proportion of spermatozoa presenting DNA fragmentation was 16.05 ± 7.39% and 35.66 ± 10.97% in fertile controls and infertile patients, respectively. Mean DNA fragmentation was significantly higher in infertile men (*p* < 0.001). Similarly, a significant difference (*p* < 0.001) was observed in the percentage of spermatozoa presenting chromatin decondensation between fertile controls and infertile men (12.91± 6.65% and 31.48 ± 9.66%, respectively) (Figure 6).

### 3.6. Correlations of Percentages of Sperm Presenting PLCζ, DNA Fragmentation, and Chromatin Decondensation with Semen Parameters

As shown in Table 3, significant correlations were observed between sperm concentration with positive PLCζ, DNA fragmentation, and chromatin decondensation (*p* = 0.043, *p* = 0.011 and *p* = 0.036, respectively). In addition, a statistically significant difference was observed for typical sperm morphology that was PLCζ-positive (*p* = 0.04) and the correlation between DNA fragmentation and viability (*p* = 0.017).

## 4. Discussion

For many years, diagnosis of male fertility has been determined essentially by sperm analysis based on semen concentration, motility, viability, and morphology. However, several patients with normal-appearing sperm parameters still undergo difficulties in achieving a successful pregnancy [16]. Indeed, other sperm parameters may have an impact on sperm competence, namely PLCζ, SCD, and DFI.

PLCζ is a soluble cytosolic sperm factor able to induce oocyte activation via the release of intracellular calcium ions from the endoplasmic reticulum. Several studies have investigated the role and function of PLCζ in male infertility as a diagnostic and prognostic biomarker candidate for fertilization failure after ICSI [17].

DNA integrity can be altered by many factors, including a defect in spermiogenesis, poor chromatin compaction, apoptosis, oxidative stress, and lifestyle [18]. Advanced paternal age is also one of the major factors causing DNA fragmentation [19,20].

The objective of this study was to examine the relationship between DNA fragmentation and chromatin decondensation and their impact on sperm function through alterations in PLCζ expression. Our analysis included 135 patients, 70 of whom experienced fertilization failure despite having normal-appearing sperm parameters. As shown in Figure 4, our data reveal a significant difference in the proportion (%) of spermatozoa expressing PLCζ, which was higher in fertile controls than in infertile patients (*p* < 0.001).

These findings are in line with recent research. Kashir et al. demonstrated that sperm PLCζ levels correlate with improved embryogenesis and pregnancy outcomes in humans and mice [21]. Azad et al. demonstrated decreased mean percentage expression of PLCζ-positive sperm in 15 patients with previous fertilization failure following ICSI [22].

A significant decrease in PLCζ expression levels was observed, especially for those who presented DNA fragmentation and chromatin decondensation greater than 30%, as presented in Figure 6. Those results are in accordance with a study conducted by Tavalaee et al. that investigated the effect of a lower percentage of spermatozoa presenting PLCζ and its correlation with a higher rate of DNA fragmentation [10].

In this study, all infertile men exhibited overall normal sperm parameters according to WHO guidelines (Table 1), with statistically significant differences observed for sperm count and morphology (*p* = 0.015 and *p* = 0.001, respectively). However, they presented abnormal DNA fragmentation and chromatin decondensation, highlighting the crucial importance of complementary sperm function tests in ensuring successful fertilization and preserving male fertility [23].

These data suggest that DNA fragmentation can impact the PLCZ1 gene [24]. The human PLCZ1 gene is composed of 15 exons situated on chromosome 12 demonstrating a genetic connection between oocyte activation deficiency (OAD) and PLCζ and thereby impacting the function and the structure of its spermatic protein [25].

Several studies, including those by Yan et al. investigated different PLCζ1 mutations in 14 samples obtained from patients with primary infertility exhibiting total or poor FF. After extracting genomic DNA from the peripheral blood and sequencing the whole exons of PLCZ1 using Sanger sequencing, 5 of the 14 patients were found to have biallelic PLCZ1 mutations in the Y and X domains, including four missense mutations, an in-frame deletion, and a splicing mutation; however, no mutations were detected in the C_2_ domain [24]. Dai et al. studied the correlation between PLCZ1 mutations and the localization of PLCζ on spermatozoa. Ten Chinese men who exhibited poor fertilization following intracytoplasmic sperm injection (ICSI), with a fertilization rate (FR) < 20%, were included in this study [26]. Three novel homozygous mutations in the PLCζ1 gene have been identified as causing FF: a nonsense variation, c.C588A (p. C196X), and two missense variants—c. T1048C (p. S350P) and c. C736T (p. L246F) [27,28].

PLCζ expression was entirely absent in spermatozoa carrying the homozygous nonsense variation p.C196X. In addition, 93.5% of spermatozoa with the p.S350P variant exhibited diffuse signals in the post-acrosomal region, while 92.6% of those with the p.L246F variant displayed signals in the equatorial region [29]. Collectively, these data suggest that PLCζ1 variations led to abnormal localization patterns of PLCζ in spermatozoa. Decreased PLCζ expression leads to abnormal Ca^2+^ oscillations and fertilization failure [30,31]. Another study investigated 37 patients presenting total or partial fertilization failure ≤ 25% after ICSI. Thirteen affected patients carried at least one mutation in each coding region of the PLCZ1 gene. Five mutations were single: p. I120M, p. R197H, p. L224P, p. H233L, and p. S500 L. The sixth mutation was caused by a deletion of two nucleotides (p. V326K fs*25). These mutations were located all over the gene: p. I120M was located at the c-terminus of the EF hand domain, which regulates calcium sensitivity, the three mutations p.R197H, p.L224P, and p.H233L were located at the X catalytic domain, which enables PLCζ to release Ca^2+^, and the mutation p.S500 L was found at C_2_, controlling PLCζ function and its degree of sensitivity [32,33].

An increase in DNA fragmentation is also associated with elevated oxidative stress, which promotes lipid peroxidation and the generation of reactive oxygen species (ROS), potentially leading to cell death and reduced sperm fertilization capacity [34]. This theory was supported by Park et al., who investigated the feasibility and reproducibility of measuring PLCζ and the relationship between PLCζ and DNA fragmentation and oxidation in human sperm. Their data showed a significant negative relationship with 8-OHdG immunoreactivity (r = −0.404, *p* = 0.009) [35].

It is therefore evident that as DNA fragmentation or chromatin decondensation increases, the expression of the spermatic protein correspondingly decreases. Since the contribution of spermatozoa to events occurring after fertilization is not limited to PLCζ, results reported here do not exclude the possibility that DNA fragmentation may influence pregnancy rates and embryonic development, thereby leading to complete fertilization failure and miscarriages [36]. Oocyte activation failure occurs when the mechanisms required to initiate embryonic development after fertilization are impaired and are distinct from oocyte maturation, which is a complex and well-organized process that includes meiotic division and recombination, nuclear maturation, and epigenetic modification. Each step of this process is regulated by a vast network of genes and every mutation can lead to recurrent failures of IVF/ICSI cycles due to poor response to ovarian stimulation, arrested oocyte maturation, poor oocyte quality, and fertilization failure.

PLCζ is a soluble factor recognized as being an essential protein for a successful fertilization process while activating several signaling pathways. A significant decrease in the protein expression for different regions on the spermatozoa was noted for infertile men. The localization of the protein in the spermatozoa is directly correlated with the success rate of fertilization and pregnancy. The characteristic localizations of PLCζ are in the equatorial, acrosome, and post-acrosomal regions of the spermatozoa or a combination of these locations. An analysis of sperm samples showed that ∼88% of PLCζ was expressed in the equatorial region, while ∼35% and ∼21% of sperm expressed the protein in the acrosomal and post-acrosomal regions, respectively [14]. Contrarily to our study, the acrosomal localization was mostly expressed by PLCζ (∼57%), followed by the equatorial localization and the combination of acrosomal and equatorial localization, as illustrated in Figure 3a–c. A significant difference was observed between the acrosomal region alone and combined acrosomal and equatorial localization according on chi-squared testing (*p* < 0.001) (Table 2).

For in vivo fertilization, spermatozoa migrate through the female reproductive tract and undergo capacitation to acquire fertilizing capability [37]. Capacitated sperm can undergo the acrosome reaction to penetrate to the zona pellucida and reach the oocyte membrane. In this condition, only the head of the spermatozoa can cross this barrier to release the cytoplasmic content and therefore acrosomal localization or a combination of acrosomal and equatorial localization can be more beneficial for successful oocyte activation, as demonstrated in this study. The importance of the acrosomal localization leading to successful fertilization has also been demonstrated in the case of globozoospermic patients, characterized by a defective morphology of the sperm head (round head) due to a DPY19-like 2 (DPY19L2) mutation that is a key causative factor related to human globozoospermia. Therefore, spermatozoa without an acrosomal bud result in oocyte activation failure [38].

Another contributing factor, advanced parental age, has been confirmed as one of the risk factors inducing male infertility. Most professional societies define this as greater than 40 years [39,40]. Advanced male age has been associated with several consequences, for example, low semen volume and alteration of sperm parameters: total sperm count, sperm motility, and normal sperm morphology. Aging can also induce gonadotropin level increase and testosterone levels decrease, resulting in a gradual modification of testicular vascularization and regular decrease in number of Sertoli and Leydig cells [40,41]. In that study, paternal age differed significantly between groups (*p* < 0.001). In our study, PLCζ protein expression was found to be independent of age, which contrasts with the findings of Junaid et al., who reported a negative correlation between advancing male age and PLCζ levels, suggesting that age could be a relevant factor when investigating PLCζ in cases of male infertility and subfertility in humans [42].

Patients with reduced semen quality due to DNA alterations may be advised to use IVF/ICSI with assisted oocyte activation (AOA) involving the artificial induction of Ca^2+^ release using ionophore A23187. An injection of complementary RNA (cRNA) or even recombinant PLCζ proteins resulted in calcium oscillations similar to those observed during fertilization and supported embryonic development up to the blastocyst stage in a mouse model. This provides evidence of the role of PLCζ as a sperm-specific factor that induces oocyte activation, Recent studies further underscored PLCζ’s significance, revealing that sperm from PLCζ-deficient (Plcz1−/−) mice failed to induce Ca^2+^ oscillations upon intracytoplasmic sperm injection (ICSI) [43]. Indeed, sperm extracts and PLCζ cRNA microinjected into the female gametes of other species can induce Ca^2+^ release. Regarding the involvement of RNA, existing theories are debatable, given that the total amount of PLCζ RNA presented in the sperm influences the concentration of calcium that is released [44,45,46]. We propose that PLCζ evaluation may serve as a future biomarker to refine the indication for AOA, but its use should currently remain investigational and complementary to established diagnostic criteria.

In this study, we observed marked variability in PLCζ expression among infertile patients, including cases of complete or near-complete absence of the protein despite normal semen parameters. This heterogeneity raises the possibility of underlying genetic variants within the PLCZ1 gene. Previous studies have reported multiple single-nucleotide polymorphisms (SNPs) and mutations in PLCZ1 that may impair its expression, function, or even localization. Although genetic analysis was not performed in the present study, targeted sequencing of PLCZ1, especially in patients with total fertilization failure and absent PLCζ on staining, could help identify mutations to explain the molecular basis of these defects. To also obtain a more detailed molecular picture of sperm DNA integrity, advanced methods such as quantification of oxidative DNA lesions, protamine 1/2 ratio analysis, and quantification of oxidative DNA lesions have been proposed. Future studies incorporating genomic screening may allow for better phenotypic stratification of male infertility and help define personalized treatment approaches, including potential indications for assisted oocyte activation.

## 5. Conclusions

In this study, we analyzed the relationships among PLCζ, SDF, and DFI. The results revealed significant correlations among these three parameters. In addition, the percentage of spermatozoa presenting SDF and DFI was significantly higher in infertile patients, indicating that sperm presenting a high degree of DNA damage was less likely able to induce oocyte activation. These results are consistent with the previous literature. Despite this result, further studies are required to assess a direct relationship between oxidative stress and the expression of PLCζ. Antioxidant therapy by administration of antioxidants and vitamins has been shown to improve DNA integrity such that sperm protein function can be recovered to increase the chances of successful fertilization. Reduced PLCζ expression and increased DNA fragmentation may contribute to fertilization failure in normozoospermic ICSI patients. While PLCζ testing shows potential to guide clinical decisions, current recommendations advise limiting AOA to confirmed oocyte activation failure. In the future, assessing PLCζ expression could help identify patients who may benefit from AOA more selectively, thus avoiding unnecessary intervention.

## Figures and Tables

**Table 1:** Sperm parameters analysis in fertile controls (n = 65) and infertile patients (n = 70).

Sperm Parameters	Fertile Controls	Infertile Patients	*p*-Value
Sperm concentration (10^6^/mL)	94.19 ± 57.77	59.97 ± 40.45	0.015 *
Progressive motility (%)	50.69 ± 11.92	42.96 ± 11.29	0.46
Viability (%)	76.58 ± 10.35	70.59 ± 9.20	0.29
Morphology (%)	11.30 ± 4.32	7.52 ± 3.24	0.001 *

Data are presented as means ± SD; * statistically significant (*p* < 0.05).

**Table 2:** Comparison of PLCζ localization between fertile controls (n = 65) and infertile patients (n = 70) using chi-squared test.

PLCζ Localization	Fertile Controls (n Positive/n Total)	Infertile Patients (n Positive/n Total)	χ^2^ (df)	*p*-Value
A	50/65	4/70	70.011 (1)	<0.001 *
Eq	16/65	9/70	1.951 (1)	0.162
A + Eq	27/65	8/70	13.691 (1)	<0.001 *

Data are presented as the number of individuals showing the presence of PLCζ localization over the total number of subjects per group. Specific localization patterns of PLCζ referred to: A: acrosomal; Eq: equatorial. The chi-squared test (df = 1) was applied to compare groups. Statistically significant differences (*p* < 0.05) are marked with asterisks.

**Table 3:** Correlations of percentage of spermatozoa presenting PLCζ, DNA fragmentation, and chromatin decondensation with sperm parameters (concentration, motility, viability, and morphology: n = 135).

	Sperm Concentration (10^6^/mL)	Progressive Motility (%)	Viability (%)	Morphology (%)
PLCζ (%)	*p* = 0.043 *	*p* = 0.4	*p*= 0.056	*p* = 0.04 *
DFI (%)	*p* = 0.011 *	*p* = 0.715	*p* = 0.017 *	*p* = 0.119
SCD (%)	*p* = 0.036 *	*p* = 0.438	*p* = 0.074	*p* = 0.219

* Significant difference *p* < 0,05). PLCζ: phospholipase C zeta; DFI: DNA fragmentation index; SCD: sperm chromatin decondensation.

## Data Availability

All the data included in this study can be obtained on request.

## Abbreviations

The following abbreviations are used in this manuscript:

ICSI	intracytoplasmic sperm injection
PLCζ	phospholipase C ζ
DFI	DNA fragmentation index
SCD	sperm chromatin decondensation
